# Improving Photocatalytic Activity for Formaldehyde Degradation by Encapsulating C_60_ Fullerenes into Graphite-like C_3_N_4_ through the Enhancement of Built-in Electric Fields

**DOI:** 10.3390/molecules28155815

**Published:** 2023-08-02

**Authors:** Dongmei Peng, Zhongfeng Zhang, Jijuan Zhang, Yang Yang

**Affiliations:** 1 College of Furniture and Art Design, Central South University of Forestry and Technology, Changsha 410000, China; pengdongmei202210@163.com (D.P.); t20050729@csuft.edu.cn (J.Z.); yang193829@163.com (Y.Y.); 2 Green Furniture Engineering Technology Research Center, National Forestry & Grassland Administration, Changsha 410004, China; 3 Green Home Engineering Technology Research Center, Changsha 410004, China

**Keywords:** graphite-like C_3_N_4_/C_60_ composite, robust built-in electric field, charge transfer, photocatalysis, formaldehyde degradation

## Abstract

The photocatalytic degradation of formaldehyde by graphite-like C_3_N_4_ is one of the most attractive and environmentally friendly strategies to address the significant threat to human health posed by indoor air pollutants. Despite its potential, this degradation process still faces issues with suboptimal efficiency, which may be attributed to the rapid recombination of photogenerated excitons and the broad band gap. As a proof of concept, a series of graphite-like C_3_N_4_@C_60_ composites combining graphite-like C_3_N_4_ and C_60_ was developed via an in situ generation strategy. The obtained graphite-like C_3_N_4_@C_60_ composites exhibited a remarkable increase in the photocatalytic degradation efficiency of formaldehyde, of up to 99%, under visible light irradiation, outperforming pure graphite-like C_3_N_4_ and C_60_. This may be due to the composites’ enhanced built-in electric field. Additionally, the proposed composites maintained a formaldehyde removal efficiency of 84% even after six cycles, highlighting their potential for indoor air purification and paving the way for the development of efficient photocatalysts.

## 1. Introduction

As the atmospheric environment continues to deteriorate, formaldehyde has emerged as a critical indoor air pollutant that poses a serious threat to human health. It is commonly discharged by industrial activities and building materials, and its impact on human health is irreversible [1,2]. Even at low concentrations, long-term exposure to formaldehyde will extremely damage the human nervous system and respiratory system. Due to growing concern over the harmful effects of formaldehyde on human health, a variety of effective strategies have been developed to eliminate it rapidly. These methods, including adsorption purification [3], thermal catalysis [4,5], biofiltration, and condensation [6,7], are commonly used in indoor air purification. However, most of these approaches involve merely adsorbing formaldehyde onto a filter medium without degrading the pollutants. As a result, these air purifiers have several drawbacks, including low adsorption capacity, the rerelease of formaldehyde into the air, and difficulties in regenerating adsorbents. Thus, it is crucial to prioritize the exploration and development of effective solutions to eliminate formaldehyde at low concentration levels, especially in indoor environments.

**Scheme 1:**
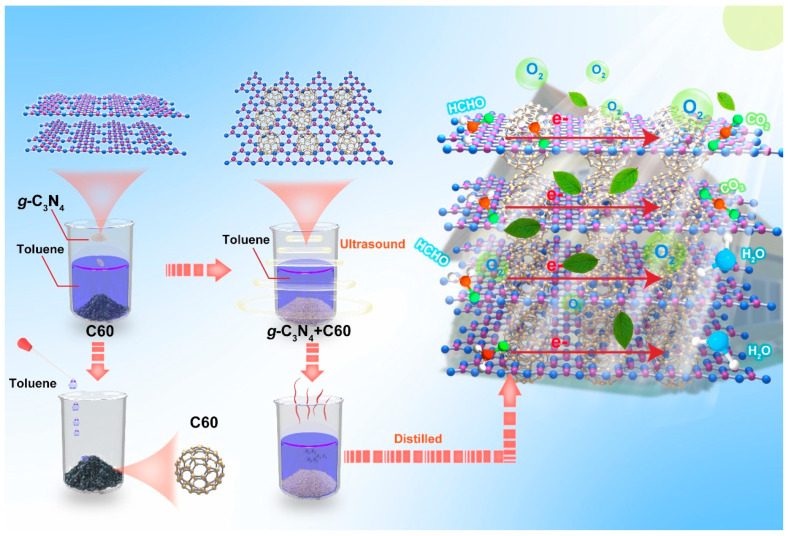
Synthetic routes and notional structures of g-C_3_N_4_ and C_60_ composites.

Photocatalytic oxidation is regarded as a versatile and promising strategy for air purification credited to its low energy consumption, high efficiency, and lack of secondary pollution; it can effectively mineralize formaldehyde to CO_2_ and H_2_O on inexpensive polymeric semiconductors under mild conditions [2,8,9,10,11,12,13]. The semiconductor graphite-like C_3_N_4_, which is considered one of the most advanced metal-free photocatalysts, has been widely applied for the photocatalytic degradation of formaldehyde due to its strong oxidation ability, low cost-effectiveness, and long-term stability [14,15,16,17,18]. However, its large band gap and limited light absorption capacity could result in the recombination of its internal excited carriers, which would undermine its photocatalytic efficiency [19,20]. To enhance the separation efficiency of photogenerated excitons, numerous strategies such as semiconductor composites [9], elemental doping [21,22], dye sensitization [23,24], surface engineering [25,26,27,28], and constructing heterojunctions [8] have been adopted to circumvent these obstacles. Despite efforts to enhance the photocatalytic efficiency of graphite-like C_3_N_4_, these strategies are limited by issues such as serious corrosion, high temperatures, and tedious preparation steps. Therefore, there is an utmost urgency to discover and develop a simple and efficient strategy for improving the photocatalytic efficiency of graphite-like C_3_N_4_ (Scheme 1).

Fullerenes, such as C_60_, are distinct forms of carbon with exceptional electronic characteristics [29,30,31,32,33,34]. C_60_ is considered favorable for efficient electron transfer reduction due to its closed-shell configuration [35,36]. The distinct structure of C_60_ makes it an outstanding electron acceptor that effectively induces quick photoinduced charge separation while experiencing comparatively slow charge recombination [37,38,39]. Wang et al. have provided a comprehensive overview of the recent notable progress in the realms of hydrogenation and oxidation facilitated by catalytic systems based on graphite-like C_3_N_4_ [40,41]. They also discovered that an amalgamation of carbon nitride and carbon nanotubes displayed a synergistic effect during optoelectronic conversion. Consequently, graphite-like C_3_N_4_ has become a category of 2D nanomaterials resembling graphite, and its distinct structure offers vast potential for utilization as a metal-free semiconductor that can govern photocatalytic reactions. Huang et al. reported that the electrical performance of covalent organic frameworks (COFs) can be enhanced by encapsulating fullerenes into the channels of COFs via a donor–acceptor interaction [42]. The improved electrical conductivity and carrier mobility can promote efficient charge transfer, which is highly desirable in photocatalytic processes.

In light of the above, using an in situ generation approach, we developed a series of photocatalytic composites, denoted as graphite-like C_3_N_4_@C_60_, wherein the guest molecule C_60_ was encapsulated into the host graphite-like C3N4 framework. The resulting graphite-like C_3_N_4_/C_60_@6:1 composite induced the formation of a strong built-in electric field, which accelerated charge transport kinetics. In addition, this composite achieved a formaldehyde degradation efficiency of up to 99% under visible-light irradiation, outperforming pure graphite-like C_3_N_4_ and C_60_. Our study presents a pioneering approach for designing photocatalysts based on graphite-like C_3_N_4_ and achieving efficient solar energy conversion.

## 2. Results and Discussion

### Characterization of the Photocatalysts

**Figure 1:**
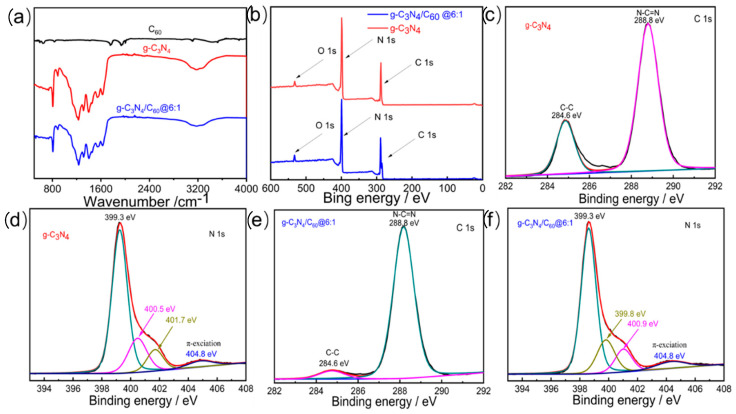
(**a**) FT-IR spectra of C_60_, g-C_3_N_4_, and g-C_3_N_4_/C_60_@6:1, obtained in transmission mode; (**b**) XPS survey spectra of spectra of C_60_, g-C_3_N_4_, and g-C_3_N_4_/C_60_@6:1; (**c**) high-resolution XPS spectra of C 1s for g-C_3_N_4_; (**d**) high-resolution XPS spectra of N 1s for g-C_3_N_4_; (**e**) high-resolution XPS spectra of C 1s for g-C_3_N_4_/C_60_@6:1; and (**f**) high-resolution XPS spectra of N 1s for g-C_3_N_4_/C_60_@6:1.

The as-prepared specimens were characterized by FT-IR spectra to confirm their structures (Figure 1a). The bands located at ~1610 cm^−1^ were ascribed to C=N stretching vibration, whereas the other strong bands, at ~1243 and ~1398 cm^−1^, were ascribed to C-N stretching vibration, matching the s-triazine ring in the graphite-like C_3_N_4_ well. The FT-IR spectrum of the C_60_ was relatively weak, but two peaks were observed at ~1760 and ~1940 cm^−1^, corresponding to the C_60′_s internal modes. There was no obvious structural variation between the graphite-like C_3_N_4_ and the 6 wt% C_60_/graphite-like C_3_N_4_ following the deposition of the C_60_. However, the characteristic peaks of the graphite-like C_3_N_4_ in the 6 wt% C_60_/graphite-like C_3_N_4_, ranging from ~1200 to 1700 cm^−1^, were shifted, indicating that there was a weak interaction between the C_60_ and the graphite-like C_3_N_4_. This may have facilitated electron transfer and improved the photocatalytic activity of the composites compared to the graphite-like C_3_N_4_.

X-ray photoelectron spectroscopy (XPS) was conducted to verify the chemical compositions and determine the valence states of different species. The graphite-like C_3_N_4_ mainly comprised the elements C, N, and O. Among them, the presence of O was attributed to H_2_O and CO_2_ absorbed on the surface of the photocatalysts. All peak positions in the XPS spectra of the graphite-like C_3_N_4_ and the 6 wt% graphite-like C_3_N_4_/C_60_ composite were calibrated using C 1s at 284.6 eV as a reference (Figure 1b). The C 1s XPS spectrum exhibited two peaks, with distinct binding energies, at 284.6 and 288.8 eV, which corresponded to C-C and N-C=N, respectively. The N 1s XPS spectrum of the graphite-like C3N4 exhibited two distinct peaks at 400.5 and 401.7 eV, which might be attributed to C=N-C and N(C)_3_, respectively, upon deconvolution. A comparison of the N 1s XPS spectrum of the graphite-like C_3_N_4_ with that of the 6 wt% graphite-like C_3_N_4_/C_60_ indicated that the binding energies of N (N-C=N) peak-shifted from 400.5 eV (graphite-like C_3_N_4_) to 399.8 eV (6 wt% graphite-like C_3_N_4_/C_60_). This shift suggests that an interaction occurred between the graphite-like C_3_N_4_ and the C_60_ (Figure 1c–f). The extremely weak peak at 404.8 eV was ascribed to π excitation. Previous studies have demonstrated that a lower binding energy of N 1s for composites is indicative of an increased electronic cloud density around the N atoms of graphite-like C_3_N_4_. This effect is attributed to intermolecular electron diffusion from conjugated polymers to the N sites of graphite-like C_3_N_4_ through intermolecular π-π interactions.

**Figure 2:**
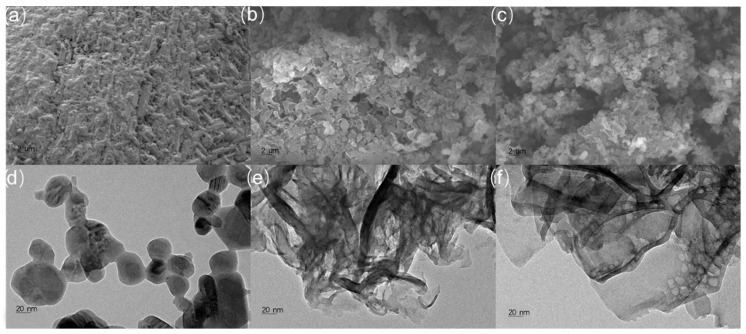
SEM images of (**a**) C_60_, (**b**) g-C_3_N_4_, and (**c**) g-C_3_N_4_/C_60_@6:1 composite and TEM images of (**d**) C_60_, (**e**) g-C_3_N_4_ and (**f**) g-C_3_N_4_/C_60_@6:1 composite.

The crystallinities for C_60_, graphite-like C_3_N_4_, and graphite-like C_3_N_4_/C_60_@6:1 were characterized with X-ray diffraction. As can be clearly observed in Figure S1, the graphite-like C_3_N_4_ and the graphite-like C_3_N_4_/C_60_@6:1 showed similar, prominent strong-intensity peaks at 25.3°, which correspond to the in-plane repeated units and the structural packing motifs of the aromatic segments, respectively, indicating the successful preparation of the graphite-like C_3_N_4_. The C_60_ displayed a cubic phase and could be indexed based on its diffraction pattern, which demonstrated two peaks, at 10.7 and 15.5, attributed to the (111) and (220) crystal planes, respectively. The deposition of C_60_ on the graphite-like C_3_N_4_/C_60_@6:1 composite surface had no remarkable effects on the structure of the graphite-like C_3_N_4_, as evidenced by the fact that the positions and intensities of the characteristic peaks at 10.7° and 15.5°, corresponding to the (111) and (220) crystal planes, respectively, remained virtually unchanged compared with those of the bare graphite-like C_3_N_4_. These observations indicate that the C_60_ was successfully deposited onto the graphite-like C_3_N_4_ without changing the crystal structure (Figure S1). The specific surface areas of the graphite-like C_3_N_4_ and the graphite-like C_3_N_4_/C_60_@6:1 were explored using adsorption–desorption isotherms performed at 77 K (Figures S2 and S3). Accordingly, the BET surface areas of the graphite-like C_3_N_4_ and the graphite-like C_3_N_4_/C_60_@6:1 were determined to be 40.0 and 29.6 m^2^/g, respectively. However, the pore volume of graphite-like C_3_N_4_/C_60_@6:1 (0.082 cm^3^/g) was considerably lower than that of graphite-like C_3_N_4_ (0.093 cm^3^/g). This difference shows that the pores of the graphite-like C_3_N_4_ were blocked or occupied by C_60_ nanoparticles. The morphology of the graphite-like C_3_N_4_/C_60_@6:1 composite was similar to that of the graphite-like C_3_N_4_, and there was no specific structure of C_60_ in the graphite-like C_3_N_4_/C_60_@6:1 composite because of its limited concentration. Moreover, the presence of dark spots with the lower transmission in the graphite-like C_3_N_4_/C_60_@6:1 composite indicated that there might have been some perturbation or disruption of the C_60_ nanoparticles. However, this perturbation did not appear to have affected the overall porous structure of the graphite-like C_3_N_4_. The small sizes of the C_60_ nanoparticles may have allowed them to be easily incorporated into the graphite-like C_3_N_4_ nanosheets, resulting in a well-dispersed composite (Figure 2).

**Figure 3:**
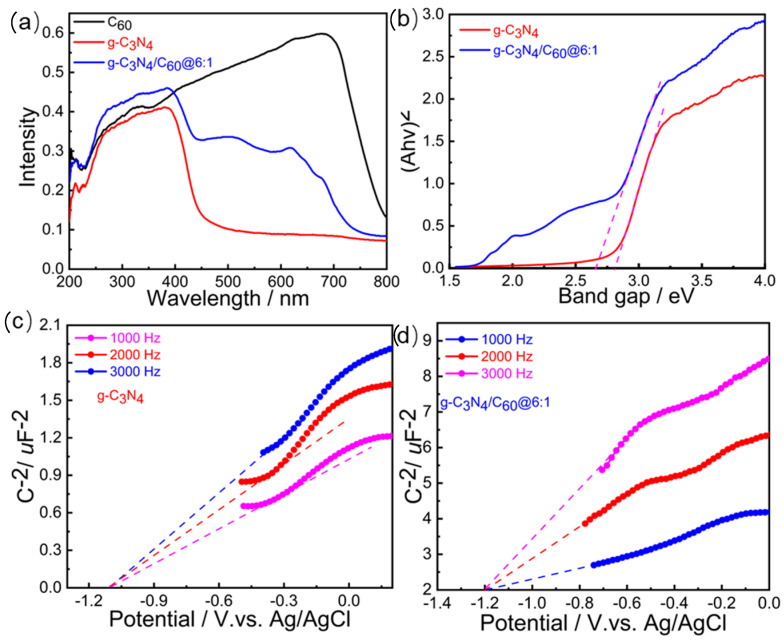
(**a**) UV-vis diffuse reflectance spectra of C_60_, g-C_3_N_4_, and g-C_3_N_4_/C_60_@6:1. (**b**) Band gaps of C_60_, g-C_3_N_4_, and g-C_3_N_4_/C_60_@6:1 determined with Kubelka-Munk-transformed reflectance. (**c**) Mott-Schottky plots of g-C_3_N_4_ in 0.2 M Na_2_SO_4_ aqueous solution at 1000 Hz, 2000 Hz, and 3000 Hz. (**d**) Mott-Schottky plots of g-C_3_N_4_/C_60_@6:1 in 0.2 M of Na_2_SO_4_ aqueous solution at 1000 Hz, 2000 Hz, and 3000 Hz.

UV-vis diffuse reflectance spectroscopy was utilized to characterize the optical properties of the C_60_, the graphite-like C_3_N_4_, and the graphite-like C_3_N_4_/C_60_@6:1. Both the C_60_ and the graphite-like C_3_N_4_/C_60_@6:1 composite exhibited wide absorption edges, at ~700 nm, ascribing to the intrinsic band gap absorption of the C_60_. (Figure 3a). The graphite-like C_3_N_4_/C_60_@6:1 composite showed obvious red-shift edges after the introduction of C_60_, indicating that the graphite-like C_3_N_4_/C_60_@6:1 composite could broaden the spectrum to the visible region compared to the bare graphite-like C_3_N_4_. Based on the equation E_g_ = 1240/λ, the band gaps of the graphite-like C_3_N_4_/C_60_@6:1 and the graphite-like C_3_N_4_ were determined to be 2.62 and 2.85 eV, respectively (Figure 3b). The smaller band gap for the graphite-like C_3_N_4_/C_60_@6:1 indicates that it is more easily excited to generate free holes and electrons. The lowest unoccupied molecular orbital (LUMO) levels of graphite-like C_3_N_4_ and graphite-like C_3_N_4_/C_60_@6:1 were calculated using the Mott−Schottky method to be −1.10 and −1.20 V versus those of Ag/AgCl, indicating that the as-prepared graphite-like C_3_N_4_/C_60_@6:1 featured a more sufficient driving force for the reduction of O_2_ to O_2_^•−^ (−0.48 V versus Ag/AgCl) compared to the graphite-like C_3_N_4_ (Figure 3c,d).

**Figure 4:**
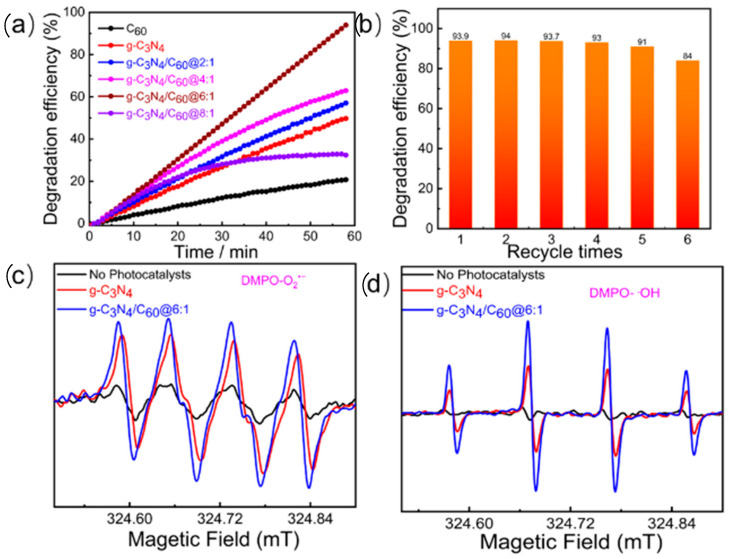
(**a**) Photocatalytic degradation efficiency of gaseous HCHO (120 ppm); (**b**) reusability of *g*-C_3_N_4_/C_60_@6:1 in gaseous HCHO; (**c**) ESR spectra of *g*-C_3_N_4_/C_60_@6:1 for detecting O_2_^•−^; and (**d**) ESR spectra of *g*-C_3_N_4_/C_60_@6:1 for detecting •OH.

The photocatalytic oxidation of formaldehyde was employed to assess the photocatalytic activity via the IR multi-gas monitor under UV-vis light irradiation for 1 h at a wavelength range of 310–800 nm and an intensity of 2.9 mW/cm^2^. As displayed in Figure 4a, the photocatalytic performance of the graphite-like C_3_N_4_/C_60_@6:1 composite in HCHO degradation varied with different mass ratios of the composite material. With an increase in the loading of the C_60_ on the graphite-like C_3_N_4_, the photocatalytic efficiency increased from 40.2% to 96.4%, indicating that the incorporation of C_60_ can remarkably enhance graphite-like C_3_N_4_ during photocatalytic HCHO degradation. As the amount of C_60_ increases in a graphite-like C_3_N_4_/C_60_ composite, it could lead to a decrease in photocatalytic activity due to shading effects that would reduce the amount of light that would reach the graphite-like C_3_N_4_. Under the experimental conditions, the photocatalytic efficiency of the C_60_ alone was found to be relatively low (14%). This observation suggests that the incorporation of C_60_ into g-C_3_N_4_ will enhance photocatalytic activity for HCHO degradation and can act as an efficient photocatalyst. Apart from its photocatalytic activity, photochemical stability is also a crucial consideration for photocatalytic materials. In the case of graphite-like C_3_N_4_/C_60_@6:1, the results of recycling experiments showed that the composite material maintained its photocatalytic activity for the oxidation of the HCHO over multiple cycles without any significant decline in activity (Figure 4b). Furthermore, the control photocatalysis experiment showed that the catalyst was needed for formaldehyde oxidation, and light irradiation alone was not sufficient to degrade the formaldehyde (not shown).

To explore the photocatalytic mechanisms of formaldehyde degradation by graphite-like C_3_N_4_/C_60_@6:1, radical detection was conducted using 5,5-dimethyl-1-pyrroline N-oxide (DMPO) as an active species-trapping agent (Figure 4c). Under visible light and dark conditions, absolute methanol was employed as a solvent to capture •OH and O_2_^•−^, respectively. Under the dark conditions, there were no characteristic EPR signals observed. However, when the sample was irradiated with the visible light, characteristic DMPO-•OH (hydroxyl radical) and DMPO-O_2_^•−^ (superoxide radical) peaks appeared. This suggests that the interaction between photoinduced electrons and holes may result in the generation of active •OH and O_2_^•−^ species. Based on the EPR experiments, the main reactive oxygen species involved in the photocatalysis were •OH and O_2_^•−^. These reactive species were produced when photoinduced charge carriers interacted with the H_2_O and O_2_ adsorbed on the surface of the catalyst. When exposed to the visible light, the photocatalyst generated photoinduced electrons, which reacted with the adsorbed O_2_ molecules to form O_2_^•−^. This radical then reacted with the H_2_O to produce •OH. Meanwhile, the surface •OH oxidized formaldehyde into formate species. The valence band holes in the catalyst directly oxidized the H_2_O and/or the −OH to form additional •OH. Eventually, the •OH further oxidized the formate species into H_2_O and CO_2_.

**Figure 5:**
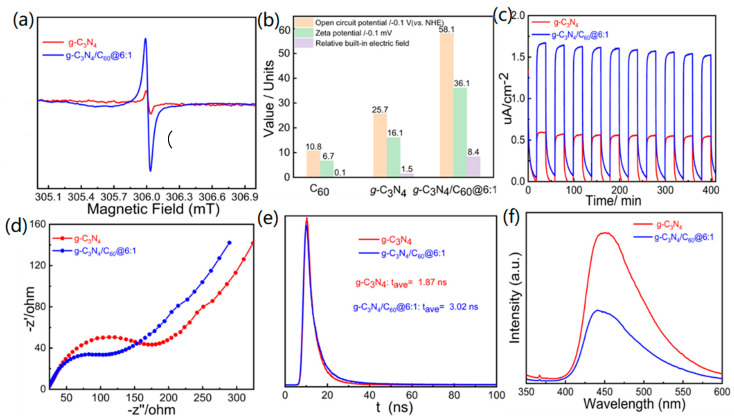
(**a**) EPR spectra for g-C_3_N_4_ and g-C_3_N_4_/C_60_@6:1; (**b**) characterization of the BEF values for C_60_, g-C_3_N_4_, and g-C_3_N_4_/C_60_@6:1; (**c**) transient photocurrent responses for g-C_3_N_4_ and g-C_3_N_4_/C_60_@6:1; (**d**) Nyquist plots for g-C_3_N_4_ and g-C_3_N_4_/C60@6:1; (**e**) photoluminescence decay traces for g-C_3_N_4_ and g-C_3_N_4_/C_60_@6:1; and (**f**) photoluminescence spectra of g-C_3_N_4_ and g-C_3_N_4_/C_60_@6:1.

In order to better understand how composites can better enhance photocatalytic activity compared to pure graphite-like C_3_N_4_, a range of photoelectrochemical properties was examined. The EPR signals of the graphite-like C_3_N_4_/C_60_@6:1 composite showed a significant increase when exposed to visible light compared with those of pure graphite-like C_3_N_4_. This indicates that these composites enable more effective production of unpaired electrons and photoinduced charge carrier pairs in graphite-like C_3_N_4_. Thus, charge migration and separation are facilitated through the encapsulation of C_60_ into pure graphite-like C_3_N_4_ (Figure 5a). The built-in electrical field is a critical factor for driving photogenerated holes and electrons to drift in reverse directions in photocatalysts, which, in turn, dramatically accelerates exciton separation. The BIEF intensities of C_60_, graphite-like C_3_N_4_, and graphite-like C_3_N_4_/C_60_@6:1 were estimated by employing the model established by Kanata, and the results indicated that BIEF strength could be assessed with both surface charge density and surface potential. The surface potentials were determined to be 10.8, 25.7, and 58.7 mV for the C_60_, the graphite-like C_3_N_4_, and the graphite-like C_3_N_4_/C_60_@6:1, respectively. Moreover, the corresponding zeta potentials of the C_60_, the graphite-like C_3_N_4_, and the graphite-like C_3_N_4_/C_60_@6:1 were −6.7, −16.1, and −36.1 V, respectively. According to the open-circuit potential and zeta potential, the graphite-like C_3_N_4_/C_60_@6:1 (8.4) had the strongest built-in electric field, which exceeded those of the C_60_ (0.1) and the graphite-like C_3_N_4_ (1.5). This significant increase in the built-in electric field indicates a strong driving force for achieving the efficient separation of excitons (Figure 5b).

The vital role of encapsulated C_60_ in the charge mobility was further explored. As expected, the graphite-like C_3_N_4_/C_60_@6:1 exhibited a higher photocurrent intensity than that of the pure graphite-like C_3_N_4_, which implied accelerated production and separation of photoinduced electron-and-hole pairs at the graphite-like C_3_N_4_/C_60_@6:1 interface (Figure 5c). Electrochemical impedance spectra were employed to explore the transfer of photogenerated charge carriers. Semicircular Nyquist plots showed a remarkable decrease in radii upon the deposition of C_60_ on graphite-like C_3_N_4_, indicating a significant enhancement in the rate of charge transfer (Figure 5d). These findings have demonstrated that the electrochemical impedance of graphite-like C_3_N_4_ is optimized when it is combined with C_60_.

Transient fluorescence was conducted to illustrate the separation behaviors of photoinduced electron-and-hole pairs by calculating the excited-state lifetimes of graphite-like C_3_N_4_ and graphite-like C_3_N_4_/C_60_@6:1. The graphite-like C_3_N_4_/C_60_@6:1 composite demonstrated a longer transient fluorescence lifetime (3.02 ns) compared to the pure graphite-like C_3_N_4_ (1.87 ns), indicating that graphite-like C3N4/C60@6:1 has a higher potential for efficiently achieving the photocatalytic degradation of gaseous HCHO (Figure 5e). A significantly lower photoluminescence intensity of the graphite-like C_3_N_4_/C60@6:1 could be observed compared with that of the graphite-like C_3_N_4_, which, in principle, suggests that graphite-like C_3_N_4_/C60@6:1 is more effective in the separation and transfer of photogenerated charge carriers and the suppression of photogenerated exciton recombination (Figure 5f).

## 3. Materials and Methods

### 3.1. Materials

Urea (98%) and C_60_ (98%) were supplied by Aladdin. Toluene and other conventional reagents were obtained from d from Beijing HWRK Chem Co., Ltd., (Beijing, China). All solvents and chemicals were used directly without any further purification.

### 3.2. Preparation of Graphite-like C_3_N_4_

With reference to the prior literature, 20 g of urea was placed in a crucible and heated to 550 °C for 5 h. The graphite-like C_3_N_4_ was obtained as powdery yellow granules and was used directly without further treatment.

### 3.3. Preparation of Graphite-like C_3_N_4_@C_60_ Composites

To prepare a mixture of C_60_ in the toluene (50 mL), the as-prepared graphite-like C_3_N_4_ (200 mg) was added. After stirring, the mixture was treated ultrasonically for 1 h. After the removal of the toluene under vacuum, the residues were rinsed with ethanol and dried to obtain gray graphite-like C_3_N_4_@C_60_ composites. Different graphite-like C_3_N_4_@C_60_ composites were synthesized, with the weight ratios of C_60_:graphite-like C_3_N_4_ ranging from 0 to 8 wt%.

### 3.4. Photocatalytic Degradation of Gaseous Formaldehyde

Approximately 300 mg of composites was evenly dispersed in 30 mL of distilled water, followed by sonication for 30 min. The resulting suspension was transferred into three surface dishes with 6 cm diameters, followed by drying at 60 °C for 12 h. The surface dishes were placed in a reactor, and the reactor was left in a closed system. Then, formaldehyde was injected using a microsyringe and the initial concentration of formaldehyde was set at about 120 ± 20 ppm. The concentrations of formaldehyde were measured online using an IR multi-gas monitor (INNOVA Air Tech Instruments Model 1412) under UV-vis light irradiation for 1 h at a wavelength range of 310–800 nm and an intensity of 2.9 mW/cm^2^.

## 4. Conclusions

In summary, various graphite-like C_3_N_4_ and C_60_ composites were developed via an in situ generation strategy. The efficiency charge separation and photocatalysis for the resultant composites were tuned by simply varying the weight ratios between the C_60_ and the graphite-like C_3_N_4_. It was found that the graphite-like C_3_N_4_/C_60_@6:1 composite exhibited the strongest built-in electric field, thus realizing efficient charge separation and rendering superior photocatalytic efficiency for formaldehyde degradation compared to the graphite-like C_3_N_4_ and the C_60._ Overall, this research has offered valuable insights into the design and development of novel synergistic systems for practical applications, particularly in the field of photocatalysis.

## Data Availability

No applicable.

## Supplementary Materials

The following supporting information can be downloaded at: https://www.mdpi.com/article/10.3390/molecules28155815/s1. Figure S1. XRD for C_60_, g-C_3_N_4_, and *g*-C_3_N_4_/C_60_@6:1. Figure S2. Nitrogen adsorption and desorption isotherms of g-C_3_N_4_, and *g*-C_3_N_4_/C_60_@6:1 at 77 K. Figure S3. pore size distribution for g-C_3_N_4_ and *g*-C_3_N_4_/C_60_@6:1.

## References

[1] Han K.H., Zhang J.S., Guo B. (2017). Toward effective design and adoption of catalyst-based filter for indoor hazards: Formaldehyde abatement under realistic conditions. J. Hazard. Mater..

[2] Liu W., Shi M., Li Y., Wu Z., Yang L., Zhang S., Xiao X., Liu C., Dai W., Chen C. (2022). Congregated-electrons-strengthened anchoring and mineralization of gaseous formaldehyde on a novel self-supporting Cu_2−x_Se/Cu_2_O heterojunction photocatalyst under visible lights: A viable mesh for designing air purifier. Appl. Catal. B Environ..

[3] Suresh S., Bandosz T.J. (2018). Removal of formaldehyde on carbon -based materials: A review of the recent approaches and findings. Carbon.

[4] Yusuf A., Snape C., He J., Xu H., Liu C., Zhao M., Chen G.Z., Tang B., Wang C., Wang J. (2017). Advances on transition metal oxides catalysts for formaldehyde oxidation: A review. Catal. Rev..

[5] Zhang Z., Jiang Z., Shangguan W. (2016). Low-temperature catalysis for VOCs removal in technology and application: A state-of-the-art review. Catal. Today.

[6] Xu Z., Qin N., Wang J., Tong H. (2010). Formaldehyde biofiltration as affected by spider plant. Bioresour. Technol..

[7] Dingle P., Tapsell P., Hu S. (2000). Reducing Formaldehyde Exposure in Office Environments Using Plants. Bull. Environ. Contam. Toxicol..

[8] Liu S.-H., Lin W.-X. (2019). A simple method to prepare g-C_3_N_4_-TiO_2_/waste zeolites as visible-light-responsive photocatalytic coatings for degradation of indoor formaldehyde. J. Hazard. Mater..

[9] Qiu S., Wang W., Yu J., Tian X., Li X., Deng Z., Lin F., Zhang Y. (2022). Enhanced photocatalytic degradation efficiency of formaldehyde by in-situ fabricated TiO_2_/C/CaCO_3_ heterojunction photocatalyst from mussel shell extract. J. Solid State Chem..

[10] Li J., Cui W., Chen P., Dong X., Chu Y., Sheng J., Zhang Y., Wang Z., Dong F. (2020). Unraveling the mechanism of binary channel reactions in photocatalytic formaldehyde decomposition for promoted mineralization. Appl. Catal. B Environ..

[11] Sheng C., Wang C., Wang H., Jin C., Sun Q., Li S. (2017). Self-photodegradation of formaldehyde under visible-light by solid wood modified via nanostructured Fe-doped WO_3_ accompanied with superior dimensional stability. J. Hazard. Mater..

[12] Shayegan Z., Lee C.-S., Haghighat F. (2018). TiO_2_ photocatalyst for removal of volatile organic compounds in gas phase—A review. Chem. Eng. J..

[13] Zhong L., Haghighat F., Lee C.-S., Lakdawala N. (2013). Performance of ultraviolet photocatalytic oxidation for indoor air applications: Systematic experimental evaluation. J. Hazard. Mater..

[14] Xiang N., Bai Y., Li Q., Han X., Zheng J., Zhao Q., Hou Y., Huang Z. (2022). ZIF-67-derived hierarchical hollow Co_3_O_4_@CoMn_2_O_4_ nanocages for efficient catalytic oxidation of formaldehyde at low temperature. Mol. Catal..

[15] Liu X., Zheng J., Peng K., Qin G., Yang Y., Huang Z. (2022). The intrinsic effects of oxygen vacancy and doped non-noble metal in TiO_2_(B) on photocatalytic oxidation VOCs by visible light driving. J. Environ. Chem. Eng..

[16] Zuo S., Zan J., Li D., Guan Z., Yang F., Xu H., Huang M., Xia D. (2021). Efficient peroxymonosulfate nonradical activity of Zn-Mn-Al_2_O_3_@g-C_3_N_4_ via synergism of Zn, Mn doping and g-C_3_N_4_ composite. Sep. Purif. Technol..

[17] Liu M., Wei C., Zhuzhang H., Zhou J., Pan Z., Lin W., Yu Z., Zhang G., Wang X. (2021). Fully Condensed Poly (Triazine Imide) Crystals: Extended π-Conjugation and Structural Defects for Overall Water Splitting. Angew. Chem. Int. Ed..

[18] Fang Y., Hou Y., Fu X., Wang X. (2022). Semiconducting Polymers for Oxygen Evolution Reaction under Light Illumination. Chem. Rev..

[19] Chen X., Weng M., Lan M., Weng Z., Wang J., Guo L., Lin Z., Qiu B. (2021). Superior antibacterial activity of sulfur-doped g-C_3_N_4_ nanosheets dispersed by Tetrastigma hemsleyanum Diels & Gilg’s polysaccharides-3 solution. Int. J. Biol. Macromol..

[20] Zhu X., Wang J., Yang D., Liu J., He L., Tang M., Feng W., Wu X. (2021). Fabrication, characterization and high photocatalytic activity of Ag–ZnO heterojunctions under UV-visible light. RSC Adv..

[21] Zhu X., Zhou Q., Xia Y., Wang J., Chen H., Xu Q., Liu J., Feng W., Chen S. (2021). Preparation and characterization of Cu-doped TiO_2_ nanomaterials with anatase/rutile/brookite triphasic structure and their photocatalytic activity. J. Mater. Sci. Mater. Electron..

[22] Patnaik S., Sahoo D.P., Parida K. (2021). Recent advances in anion doped g-C_3_N_4_ photocatalysts: A review. Carbon.

[23] Qin J., Huo J., Zhang P., Zeng J., Wang T., Zeng H. (2016). Improving the photocatalytic hydrogen production of Ag/g-C_3_N_4_ nanocomposites by dye-sensitization under visible light irradiation. Nanoscale.

[24] Yang L., Bai X., Shi J., Du X., Xu L., Jin P. (2019). Quasi-full-visible-light absorption by D35-TiO_2_/g-C3N4 for synergistic persulfate activation towards efficient photodegradation of micropollutants. Appl. Catal. B Environ..

[25] Yu Y., Yan W., Wang X., Li P., Gao W., Zou H., Wu S., Ding K. (2018). Surface Engineering for Extremely Enhanced Charge Separation and Photocatalytic Hydrogen Evolution on g-C_3_N_4_. Adv. Mater..

[26] Liu D., Chen D., Li N., Xu Q., Li H., He J., Lu J.-M. (2020). Surface Engineering of g-C_3_N_4_ by Stacked BiOBr Sheets Rich in Oxygen Vacancies for Boosting Photocatalytic Performance. Angew. Chem. Int. Ed..

[27] Liu H., Zhang Z., Xu X., Zhou Y., Liu J., Yang H. (2022). Rational design via surface engineering on dual 2-dimensional ZnSe/g-C_3_N_4_ heterojunction for efficient sequestration of elemental mercury. Chem. Eng. J..

[28] Talukdar M., Behera S.K., Bhattacharya K., Deb P. (2019). Surface modified mesoporous g-C_3_N_4_@FeNi_3_ as prompt and proficient magnetic adsorbent for crude oil recovery. Appl. Surf. Sci..

[29] Clancy A., Bayazit M.K., Hodge S.A., Skipper N.T., Howard C.A., Shaffer M.S.P. (2018). Charged Carbon Nanomaterials: Redox Chemistries of Fullerenes, Carbon Nanotubes, and Graphenes. Chem. Rev..

[30] Paquin F., Rivnay J., Salleo A., Stingelin N., Silva C. (2015). Multi-phase semicrystalline microstructures drive exciton dissociation in neat plastic semiconductors. J. Mater. Chem. C.

[31] Tezuka N., Umeyama T., Matano Y., Shishido T., Yoshida K., Ogawa T., Isoda S., Stranius K., Chukharev V., Tkachenko N.V. (2011). Photophysics and photoelectrochemical properties of nanohybrids consisting of fullerene-encapsulated single-walled carbon nanotubes and poly(3-hexylthiophene). Energy Environ. Sci..

[32] Huber R.C., Ferreira A.S., Thompson R., Kilbride D., Knutson N.S., Devi L.S., Toso D.B., Challa J.R., Zhou Z.H., Rubin Y. (2015). Long-lived photoinduced polaron formation in conjugated polyelectrolyte-fullerene assemblies. Science.

[33] Cai W., Chen C.-H., Chen N., Echegoyen L. (2019). Fullerenes as Nanocontainers That Stabilize Unique Actinide Species Inside: Structures, Formation, and Reactivity. Accounts Chem. Res..

[34] Zieleniewska A., Lodermeyer F., Roth A., Guldi D.M. (2018). Fullerenes—How 25 years of charge transfer chemistry have shaped our understanding of (interfacial) interactions. Chem. Soc. Rev..

[35] Chen X., Chen H., Guan J., Zhen J., Sun Z., Du P., Lu Y., Yang S. (2017). A facile mechanochemical route to a covalently bonded graphitic carbon nitride (g-C_3_N_4_) and fullerene hybrid toward enhanced visible light photocatalytic hydrogen production. Nanoscale.

[36] Lee H.K.H., Telford A.M., Röhr J.A., Wyatt M.F., Rice B., Wu J., Maciel A.d.C., Tuladhar S.M., Speller E., McGettrick J. (2018). The role of fullerenes in the environmental stability of polymer:fullerene solar cells. Energy Environ. Sci..

[37] Li P., Fang J., Wang Y., Manzhos S., Cai L., Song Z., Li Y., Song T., Wang X., Guo X. (2021). Synergistic Effect of Dielectric Property and Energy Transfer on Charge Separation in Non-Fullerene-Based Solar Cells. Angew. Chem. Int. Ed..

[38] Huang Q., Zhuang G., Jia H., Qian M., Cui S., Yang S., Du P. (2019). Photoconductive Curved-Nanographene/Fullerene Supramolecular Heterojunctions. Angew. Chem..

[39] Eom T., Barát V., Khan A., Stuparu M.C. (2021). Aggregation-free and high stability core–shell polymer nanoparticles with high fullerene loading capacity, variable fullerene type, and compatibility towards biological conditions. Chem. Sci..

[40] Zhang P., Deng J., Mao J., Li H., Wang Y. (2015). Selective aerobic oxidation of alcohols by a mesoporous graphitic carbon nitride/N-hydroxyphthalimide system under visible-light illumination at room temperature. Chin. J. Catal..

[41] Gong Y., Li M., Li H., Wang Y. (2015). Graphitic carbon nitride polymers: Promising catalysts or catalyst supports for heterogeneous oxidation and hydrogenation. Green Chem..

[42] Xu X., Yue Y., Xin G., Huang N. (2022). Rational Construction of Electrically Conductive Covalent Organic Frameworks through Encapsulating Fullerene via Donor–Acceptor Interaction. Macromol. Rapid Commun..

