# Supplementary material for: Improving Photocatalytic Activity for Formaldehyde Degradation by Encapsulating C60 Fullerenes into Graphite-like C3N4 through the Enhancement of Built-in Electric Fields
Source: Molecules. 2023 Aug 2;28(15):5815. doi: 10.3390/molecules28155815 (PMC10420677; doi:10.3390/molecules28155815)
Supplement: Supplementary file 1 [file molecules-28-05815-s001.zip › molecules-2476327-supplementary.pdf]

# *Supporting information*

# Improving Photocatalytic Activity for Formaldehyde Degradation by Encapsulating $C_{60}$ Fullerenes into Graphite-like $C_3N_4$ through the Enhancement of Built-in Electric Fields

**Dongmei Peng [1,2,3], Zhongfeng Zhang [1,2,3,*], Jijuan Zhang [1,2,3] and Yang Yang [1,2,3]**

1 College of Furniture and Art Design, Central South University of Forestry and Technology, Changsha 410000, China; pengdongmei202210@163.com (D.P.); t20050729@csuft.edu.cn (J.Z.); yang193829@163.com (Y.Y.)
2 Green Furniture Engineering Technology Research Center, National Forestry & Grassland Administration, Changsha 410004, China
3 Green Home Engineering Technology Research Center, Changsha 410004, China
* Correspondence: t19990735@csuft.edu.cn

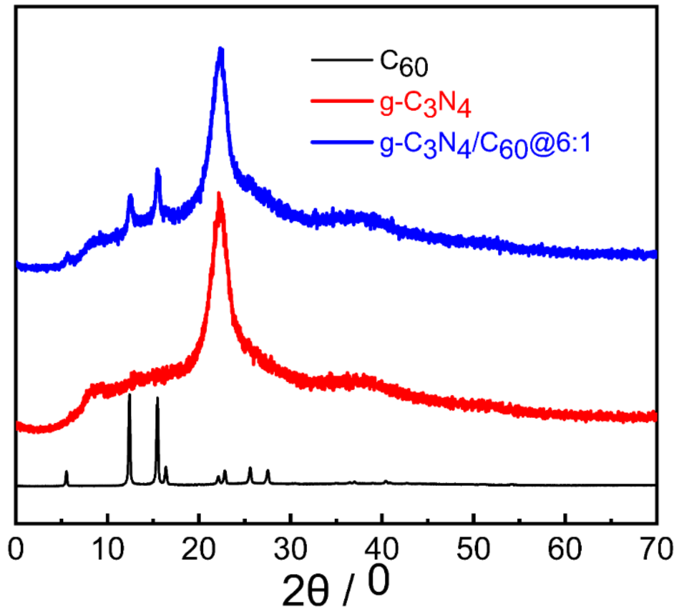


**Figure S1**. XRD for $C_{60}$, g-$C_3N_4$, and g-$C_3N_4$/$C_{60}$@6:1.

These observations indicate that $C_{60}$ was successfully deposited onto g-$C_3N_4$ without changing the crystal structure (Figure S1).

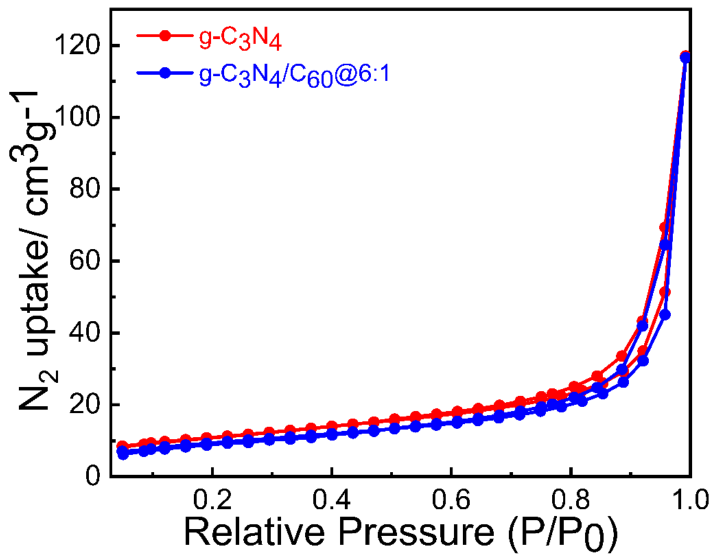


**Figure S2**. Nitrogen adsorption and desorption isotherms of g-$C_3N_4$, and *g*-$C_3N_4$/$C_{60}$@6:1 at 77 K.

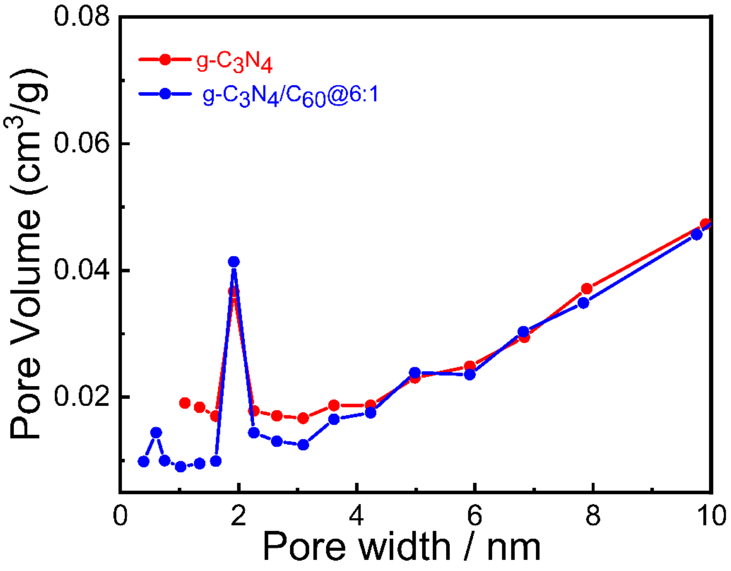


**Figure S3**. pore size distribution for g-$C_3N_4$ and *g*-$C_3N_4$/$C_{60}$@6:1.
